# Maintaining Program Fidelity in a Changing World: National Implementation of a School-Based HIV Prevention Program

**DOI:** 10.1007/s11121-023-01614-1

**Published:** 2023-11-18

**Authors:** Elizabeth Schieber, Lynette Deveaux, Lesley Cotrell, Xiaoming Li, Stephenie C. Lemon, Arlene S. Ash, Karen MacDonell, Samiran Ghosh, Maxwell Poitier, Glenda Rolle, Sylvie Naar, Bo Wang

**Affiliations:** 1 Department of Population and Quantitative Health Sciences, University of Massachusetts Chan Medical School, 368 Plantation Street, Worcester, MA 01605 USA; 2 Office of HIV/AIDS, Ministry of Health, Shirley Street, Nassau, Bahamas; 3 Department of Pediatrics, West Virginia University, 959 Hartman Run Road, Morgantown, WV 26506 USA; 4 Department of Health Promotion, Education, and Behavior, University of South Carolina Arnold School of Public Health, 915 Greene Street, Columbia, SC 29208 USA; 5 Center for Translational Behavioral Science, Florida State University College of Medicine, 2010 Levy Ave. Building B, Tallahassee, FL 32310 USA; 6 Department of Biostatistics and Data Science, School of Public Health, The University of Texas, 1200 Pressler Street, Houston, TX 77030 USA

**Keywords:** Program fidelity, Implementation dose, Evidence-based intervention, HIV prevention

## Abstract

Large-scale, evidence-based interventions face challenges to program fidelity of implementation. We developed implementation strategies to support teachers implementing an evidence-based HIV prevention program in schools, Focus on Youth in The Caribbean (FOYC) and Caribbean Informed Parents and Children Together (CImPACT) in The Bahamas. We examined the effects of these implementation strategies on teachers’ implementation in the subsequent year after the initial implementation during the COVID-19 pandemic. Data were collected from 79 Grade 6 teachers in 24 government elementary schools. Teachers completed training workshops and a pre-implementation questionnaire to record their characteristics and perceptions that might affect their program fidelity. School coordinators and peer mentors provided teachers with monitoring, feedback, and mentoring. In Year 1, teachers on average taught 79.3% of the sessions and 80.8% of core activities; teachers in Year 2 covered 84.2% of sessions and 72.9% of the core activities. Teachers with “good” or “excellent” school coordinators in the second year taught significantly more sessions on average (7.8 vs. 7.0, *t* = 2.04, *P* < 0.05) and more core activities (26.3 vs. 23.0, *t* = 2.41, *P* < 0.05) than teachers with “satisfactory” coordinators. Teachers who had a “good” or “satisfactory” mentor taught more sessions than teachers who did not have a mentor (7.9 vs. 7.3; *t* = 2.22; *P* = 0.03). Two-level mixed-effects model analysis indicated that teachers’ program fidelity in Year 1, confidence in the execution of core activities, and school coordinators’ performance were significantly associated with Year 2 implementation dose. Implementation of FOYC + CImPACT was significantly associated with improved student outcomes. Teachers maintained high fidelity to a comprehensive HIV prevention program over 2 years during the COVID-19 pandemic. Future program implementers should consider additional implementation support to improve the implementation of school-based programs.

**Supplementary Information:**

The online version contains supplementary material available at 10.1007/s11121-023-01614-1.

## Introduction

Not all evidence-based interventions (EBI) live up to their potential in real-world settings (Chambers et al., 2013). EBIs are prevention programs with documented evidence-based effectiveness for producing the desired outcomes within a particular area (Hailemariam et al., 2019). The successful translation of EBIs to applied settings is potentially affected by several factors associated with implementing the program: the new context, audience receptivity, or larger contextual factors such as policies or other events (Shelton et al., 2018). A review of the literature estimated that two-thirds of EBI implementations fail to achieve expectations or previously established outcomes (Cook et al., 2019). This points to a need to examine how best to transition an EBI into real-world school settings. Further, even less is known about how well or under what conditions programs are sustained with fidelity (Moullin et al., 2020). To ensure continued implementation of EBIs, we must identify factors associated with successful implementation, recognizing that, to remain successful over time, a program may require adequate support for facilitators and modifications to the real-world context (Herlitz et al., 2020). Program fidelity, i.e., the extent to which the intervention is executed as intended, can affect the degree to which desired program outcomes are achieved. Barbee et al. (2021) summarized five constructs that contribute to fidelity: adherence to the program as designed, the dosage or amount of a program experienced by participants, the quality of intervention delivery, participants’ responsiveness and engagement with program activities, and differentiation from treatment as usual. Poor fidelity with any of these components may affect the expected EBI outcomes, such as participants’ gains in knowledge and skills, which wastes time and resources (Barbee et al., 2021).

### Implementation of School-Based EBIs

Implementation of EBIs within school settings has been less than optimal (Cook et al., 2019). Herlitz et al. (2020) conducted a systematic review of 18 school-based EBIs that had been evaluated years after rollout. No EBI was sustained completely, meaning that the level of program fidelity observed during initial implementation trials was not maintained across multiple school years. Factors noted as being associated with the limited implementation of these programs included staff and teachers’ commitment, motivation, and self-efficacy, a reduction in mental resources (e.g., continued training), other curriculum priorities, lack of funding, a changing school climate, and the intervention structure (i.e., how well the EBI fit into existing curriculum and school policies; Herlitz et al., 2020). Long delays between implementing EBI procedures (e.g., between school years) may also threaten fidelity, since learning decays over time. van Nassau et al. (2016) observed this when teachers presenting an obesity program delivered 58% of the lessons in Year 1 and only 37% of the lessons in the following year. Similarly, 78% of teachers initially implementing a substance use prevention program covered the majority of the curriculum, but by the second year, only 25% of teachers covered the curriculum as intended (Rohrbach et al., 1993). Although longitudinal data on the implementation of school-based EBI are limited, achieving and maintaining high fidelity is a well-recognized issue in implementation science (Proctor et al., 2015).

### Training, Mentorship, and Feedback to Increase Program Fidelity

Achieving the desired outcomes of an EBI requires adequate training and support for program staff (e.g., teachers) to implement the program as designed (Moir, 2018). Moir noted that several *competency drivers* might support initial and continued implementation by ensuring that staff are prepared to deliver EBIs: staff selection, training, consultation/coaching, and staff performance evaluation. Ensuring the competency of staff prior to EBI rollout is important for successful implementation, but it may not suffice to ensure long-term fidelity; long-term implementation of EBI may also require resources for continued monitoring of implementation and training refreshers (Herlitz et al., 2020). Although staff turnover and changing curriculum requirements are threats to program delivery, annual training for new teachers and refreshers for returning teachers, mentorship, and monitoring can mitigate these threats (Bastable et al., 2020; Clayton et al., 2018). For example, explicit training on sexual health supports teachers’ self-efficacy for teaching sexual education (Clayton et al., 2018) and annual trainings also support school-based EBI implementation across school years (Wang et al., 2015). Ongoing feedback helps maintain fidelity by identifying implementation issues and allowing timely correction (Kershner et al., 2014). Coaching and mentorship, particularly modeling the skills used in the program and sharing data reports and feedback, can also increase program fidelity (Bastable et al., 2020). Coaching and mentorship can pair teachers with peers who understand implementation barriers and can assist with strategies to ensure fidelity. This provides additional support to facilitators and creates a fidelity monitoring system.

### Opportunity to Focus on School-Based EBI Implementation

There is a rich literature on school-based interventions to reduce HIV transmission among individuals in grades 7 through 12 (Chin et al., 2012; Denford et al., 2017; Fonner et al., 2014; Mavedzenge et al., 2014). Group- and school-based sex education interventions are initially effective in decreasing risky adolescent behavior and sexual activity with the goals of preventing pregnancy, sexually transmitted infections, and HIV/AIDS. *Focus on Youth in the Caribbean* (FOYC) plus *Caribbean Informed Parents and Children Together* (CImPACT) is an EBI designed to teach adolescents safe sexual skills (e.g., condom use) and to encourage reductions in risky behaviors (e.g., substance use, delinquency, and sexual risks). FOYC and CImPACT were adapted from FOY + ImPACT, which has been identified as a best-evidence intervention for HIV prevention by the CDC’s “Diffusion of Effective Behavioral Interventions (DEBI)” program (CDC, 2020; Collins Jr & Sapiano, 2016).

HIV prevalence in The Bahamas was high (reaching 4% in the late 1990s), and new HIV diagnoses are observed as early as the 15–24 age group (National HIV/AIDS Programme, 2018). To reach as many Bahamian youth as possible, and at an age prior to when most students become sexually active, the government decided to adapt an EBI to The Bahamas through public schools (Deveaux et al., 2011). Longitudinal evaluations of FOYC + CImPACT showed that the intervention significantly increased Bahamian youths’ HIV/AIDS knowledge, perceptions of their ability to use condoms, condom-use intention, and actual condom use (Chen et al., 2010; Gong et al., 2009). FOYC + CImPACT has been delivered as part of the Health and Family Life Education (HFLE) curriculum and in parent-teacher meetings since 2011.

During our previous (2011–2016) standard implementation (teacher training only) period (2011–2016), teachers taught slightly over half of the sessions (Wang et al., 2015), which is consistent with other school-based EBI implementations (Cook et al., 2019). In the current study, we sought to increase teachers’ program fidelity by providing teachers with biweekly monitoring and feedback (BMF) and site-based assistance and mentorship (SAM) (Wang et al., 2022).

These implementation strategies were evaluated using a multiphase optimization strategy (MOST, Collins et al., 2005) design and were found to be effective in supporting teachers’ program fidelity (Wang et al., 2022). The national implementation study was guided by the Exploration, Preparation, Implementation, Sustainment (EPIS) model (Aarons et al., 2011). EPIS describes elements of leadership, staffing, fidelity monitoring, and support that we have built into our implementation methods, which are also in line with Moir’s (2018) competency drivers. Extensive research over the past decade has examined the fidelity of initial EBI implementation. Fewer publications have examined the factors that influence EBI implementation after the initial rollout. Using data from the ongoing national implementation study of FOYC + CImPACT in The Bahamas, we explored (1) the extent to which program fidelity was maintained 1 year following the initial rollout (i.e., how much of the intervention curriculum the teachers delivered to the next cohort of students) and (2) what teacher- and school-level factors influenced teachers’ program fidelity and implementation dose in the subsequent year.

## Method

### Study Site

The 24 elementary schools in New Providence in The Bahamas participated in the first wave of national implementation of FOYC + CImPACT in the 2019–2020 school year (Year 1). Implementation continued through 2020–2021 (Year 2). All Grade 6 teachers who teach the Health and Family Life Education (HFLE) curriculum were invited to participate in this study. There were 79 teachers from 24 schools in New Providence in total (over 90% of HFLE teachers consented to this project). Seventy-nine Grade 6 teachers participated in the study; 70 teachers taught in both Years 1 and 2 (i.e., nine teachers left their positions and were replaced for Year 2).

### Program Implementation

The combined FOYC + CImPACT intervention included teacher-delivered components targeted toward students and their parents. The 8-session FOYC adolescent HIV prevention curriculum (with 30 core activities) was offered to Grade 6 students with annual booster sessions from Grades 7 through 9 during HFLE classes. The single CImPACT parental monitoring session (with 5 core activities) was incorporated into parent-teacher meetings. Teachers received additional support while they implemented the program including BMF from school coordinators and SAM from peer mentors (Wang et al., 2022), to improve their program delivery and attempt to continue the program fidelity over time.

### Teacher Training

Teachers were invited to attend an annual training workshop for FOYC + CImPACT. Three Bahamian FOYC trainers and a US training specialist with extensive experience with FOYC + CImPACT led the 2-day training workshop for 61 (77.2%) Grade 6 HFLE teachers in Fall 2019. The training followed FOY guidelines and consisted of clear objectives, short lectures, interactive group discussions, videos, demonstrations of curricula and skills, skill practice, role play, and teach-backs (Lauer et al., 2014). Because of the COVID-19 pandemic, the 2020 training workshop took place virtually and featured videos of six high-performing experienced Grade 6 HFLE teachers modeling FOYC + CImPACT activities. A total of 57 (72.2%) teachers attended the 2020 Year 2 virtual webinar. The purpose of the workshop and webinar was to increase teachers’ curriculum knowledge, strengthen teachers’ attitudes about the positive effects of the curriculum, and improve teachers’ skills and comfort with the curriculum. Specifically, teachers (1) reviewed the need for HIV prevention in The Bahamas, (2) received an overview of FOYC + CImPACT and its past effectiveness, (3) observed models of the 35 core activities and walked through the eight sessions of FOYC, (4) participated in a didactic question-and-answer period about menstruation and contraception and condom use, and (5) observed a model of CImPACT. Teachers also received guidance toward strategies to implement the curriculum in their classrooms as well as copies of the FOYC teacher training manual and FOYC + CImPACT 24/7 flash drives for “point-of-care” guidance.

### School Coordinators and Peer Mentors

A school coordinator was identified for each of the 24 schools to complete the BMF. They captured teachers’ implementation and progress over the course of the program period. School coordinators also reported any implementation issues to researchers in New Providence. SAM was also available to teachers implementing the curriculum during this time. Twelve high-performing teachers acted as mentors for at-risk and moderate-performing teachers. They were trained to identify challenges, assist teachers in preparing for sessions, and provide guidance for curriculum delivery. Two Bahamian trainers with extensive experience with FOYC + CImPACT conducted 2- to 3-h training sessions with school coordinators and mentors each year.

### Measures

#### Program Delivery Quality

Implementation dose was defined as the number of sessions completed (from a total of 9 sessions) or the number of the 35 core activities of the FOYC + CImPACT curriculum taught by teachers. Teachers also recorded how many core activities were taught exactly as outlined in the manual. After each session, teachers completed a Teacher Implementation Checklist specific to the individual FOYC and CImPACT sessions (i.e., there were specific core activities assigned to each session’s topic). They documented the activities covered in each session. Trained observers attended 10% of teachers’ sessions and completed the checklist to determine agreement between teachers’ and observers’ reports.

#### Teachers’ Characteristics, Training Experience, and Perceptions

In Year 1 (and in Year 2 for the nine new teachers), teachers completed a pre-implementation questionnaire to provide information known to influence program fidelity: their level of formal education, years as a teacher, attendance at the training workshop, their perceptions of the importance of HIV prevention for Grade 6 students (very meaningful, somewhat meaningful, or not at all meaningful), their comfort level teaching the FOYC + CImPACT curriculum, and any competing lessons or teaching priorities. The pre-implementation questionnaire used a 5-point Likert scale (1 = “totally disagree” to 5 = “totally agree”) and consisted of four items assessing teachers’ perceived *principal supportiveness* (e.g., “My principal expects FOYC to be taught to 6th graders.”) (Battistich et al., 1997; Liu et al., 2014); five items assessing teachers’ *confidence* teaching five topics such as condom use, teen pregnancy, and HIV/AIDS (e.g., “I feel like I can complete teaching the whole FOYC program.”) (Rijsdijk et al., 2014); and eight items assessing *attitudes toward sex education* in schools (e.g., “School is the ideal place to teach sex education as one of the many different aspects of education.”) (Martínez et al., 2014). The internal consistency (Cronbach’s *α*) of the scales is adequate (principal supportiveness *α* = 0.85; confidence, *α* = 0.91; attitudes toward sex education, *α* = 0.68).

#### Strategy Delivery Quality

The national school coordinator, New Providence school coordinator, and The Bahamas FOYC project manager, as well as FOYC curriculum trainers, assessed the performances of school coordinators and mentors with a brief 14-item questionnaire annually. Evaluators ranked school coordinators’ overall performances as “unsatisfactory,” “satisfactory,” “good,” or “excellent,” on several items including their knowledge about teachers’ implementation activities, communication with the FOYC research office, and submission of required materials. Mentors were also rated for their performance, and the number of mentoring sessions they held in the last academic year was noted. Assessors also took free response notes of school coordinators’ and mentors’ performances.

#### Student Outcomes

Students completed an anonymous curricular assessment instrument adapted by the MOE from a version of the Bahamian Youth Health Risk Behavioral Inventory (Deveaux et al., 2011). They first completed the assessment prior to experiencing FOYC + CImPACT in Grade 6 and repeated it at the beginning of Grade 7 (the follow-up was originally scheduled at the end of Grade 6 but was delayed by the COVID-19 pandemic). The instrument assessed HIV/AIDS knowledge, preventative reproductive health skills, and some perceptions of self-efficacy, intentions, and self-reported behaviors. HIV/AIDS knowledge was assessed via 16 true or false statements with correct responses scored 1 and incorrect 0, for a maximum knowledge score of 16. Preventative reproductive health skills were assessed via an adaptation of the Condom-use Skills Checklist (Stanton et al., 2009), which used six true or false statements that describe correct condom use (e.g., “when people use a condom, the condom is unrolled before it goes on”) with correct responses scored 1 and incorrect 0 for a maximum skill score of 6. Self-efficacy of students’ ability to practice safe sex skills was assessed via a five-item, 5-point Likert scale (e.g., “I could get condoms;” 1 = “strongly disagree” to 5 = “strongly agree”); the internal consistency of the scale was 0.78. A mean across the five items (range 1 to 5) was used to create a composite score. Intention to use condoms for protection was assessed on a 5-point Likert scale (1 = “no chance in the world” to 5 = “yes, big chance that I would”) for the question, “what are the chances that you would use a condom if you need to prevent yourself from getting HIV?”.

### Analysis

We calculated the frequency distributions of the number of sessions taught and the number of core activities taught during the initial implementation in 2019–2020 and implementation in 2020–2021. Histograms graphically displayed teachers’ implementation of the FOYC + CImPACT intervention in Years 1 and 2. To identify factors associated with teachers’ implementation in Year 2, we used a one-way analysis of variance and two-sample *t*-tests to compare the number of core activities (from a total of 35 activities) and the number of sessions taught (from among a total of 9 sessions) by teachers according to teacher characteristics, training and teaching experience, and performance of school coordinators and mentors. We used Pearson’s (for continuous variables) and Spearman’s (for ordinal variables) correlations to examine the associations between implementation dose and factors influencing teachers’ implementation. The responses to anonymous student assessments were aggregated at the classroom level and linked to the teachers’ data.

We used linear mixed-effects models to examine the association between implementation strategies (i.e., BMF and SAM) and teachers’ continued implementation (number of sessions and core activities taught). School random effects were included in the mixed models because teachers are clustered within schools. We included other variables that were found to be significant in our bivariate analysis and those that have been shown to be significant factors in the existing literature (e.g., perceived principal support, confidence). All the variables are at the teacher level (except school coordinators and mentors’ performance at the school-level covariates). There are 79 teachers who are clustered within 24 schools. Student data were aggregated by classroom at the teacher level. A two-level mixed-effects linear regression model used in this analysis can be described by ${Y}_{ij}=X\beta +{u}_{i}+{e}_{ij},$ where ${Y}_{ij}$ denotes the outcome of interest from the *i*th school ($i=1,\dots ,24$) and* j*th teacher in the *i*th school ($j=1,\dots ,{n}_{i}$, with ${\sum }_{i=1}^{24}{n}_{i}=79$), $\beta$ denotes the vector of fixed effects for the covariates, ${u}_{i}$ is the random effect due to school, and ${u}_{i}{\perp e}_{ij}$ are mutually independent with normal distribution each. SAS PROC MIXED is used which has likelihood-based and restricted likelihood-based statistics implemented. All statistical analyses were performed using the SAS 9.4 statistical software package (SAS Institute Inc., Cary, NC, USA).

## Results

### Teachers’ Implementation of FOYC + CImPACT in Year 1 and Year 2

**Fig. 1:**
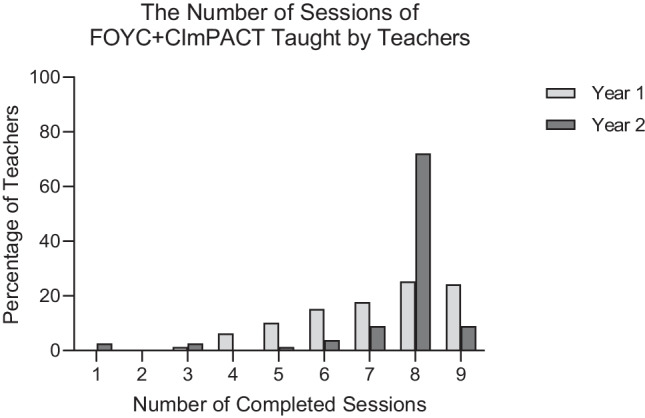
Histogram of the percentage of 79 Grade 6 teachers who covered each number of FOYC + CImPACT sessions in 2 years of implementation

**Fig. 2:**
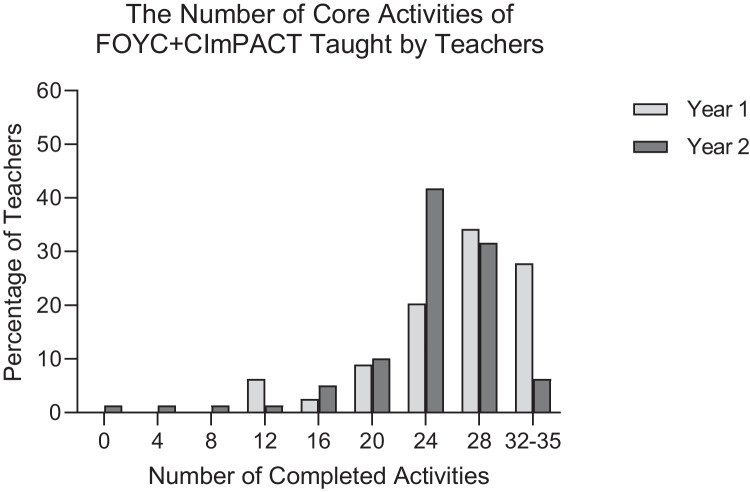
Histogram of the percentage of 79 Grade 6 teachers that covered various numbers of core activities of FOYC + CImPACT in 2 years of implementation

Figure 1 depicts the proportion of teachers that taught each of the nine sessions of FOYC + CImPACT in Years 1 and 2 of implementation. On average, teachers covered 7.14 of 9 sessions (79.3%) in Year 1 and 7.58 (84.2%) in Year 2. Individual teachers completed more sessions in Year 2 of implementation, with 81.0% of teachers covering at least 8 sessions, whereas only 49.4% of teachers in Year 1 covered at least 8 sessions. Only 8.9% of teachers covered all 9 sessions in Year 2 (down from 24.1% in Year 1), and fewer were able to host the CImPACT session in Year 2, because of the COVID-19 pandemic. Figure 2 shows the proportion of teachers that covered various levels of the core activities identified for FOYC + CImPACT. On average, teachers covered more activities in Year 1 (80.9% of activities) than in Year 2 (72.9%) activities. More teachers covered 32–35 of the core activities in Year 1 than in Year 2 (27.8% and 6.3%, respectively). The activities skipped most often in Year 2 were interactive group activities and games that worked best with in-person engagement. For example, an HIV transmission game was not taught by 17% of teachers in Year 1 and 43% of teachers in Year 2, and a game meant to simulate alcohol intoxication was skipped by 16% of teachers in Year 1 and 47% in Year 2.

### Bivariate Association of Teachers’ Characteristics, Training Experience, Perceptions, and Teacher’s Implementation in Year 2

Table 1 summarizes the relations between the teachers’ level of education and years spent teaching, experience with FOYC + CImPACT training, perceptions of the importance of FOYC + CImPACT for their students, the performances of their school coordinators and mentors, and teachers’ implementation dose and program fidelity. The variable with the largest, significant relationship with the number of sessions, core activities taught, and core activities taught according to the manual was the performance of the school coordinators. School coordinators whose performance was rated as “good” or “excellent,” as opposed to those that rated the performance as “satisfactory,” taught on average more sessions (7.8 vs. 7.0, *t* = 2.04, *P* < 0.05), covered more core activities (26.3 vs. 23.0, *t* = 2.41, *P* < 0.05), and covered more activities exactly as described in the manual (20.9 vs. 16.5, *t* = 2.31, *P* < 0.05). Teachers with a “good” or “satisfactory” mentor taught on average more sessions than those without a mentor (7.9 vs. 7.3, *t* = 2.22, *P* < 0.05). Teachers with other teaching priorities were significantly more likely to cover core activities exactly as outlined in the manual (22.2 vs. 18.8, *t* = 2.21, *P* < 0.05).

**Table 1:** Association between teacher’s characteristics, training experience, performance of their school coordinators and mentors, and number of core activities and sessions taught in the classroom among 79 Grade 6 teachers in the subsequent year after the initial implementation

Variables	Percent of teachers	Number of core activities taught (0–35)	Number of core activities taught exactly as outlined (0–35)	Number of sessions taught (0–9)
		Mean (SD)	Mean (SD)	Mean (SD)
Years as teacher or guidance counselor				
1 ~ 5 years	15.1	27.1 (3.8)	22.6 (5.9)	8.1 (0.4)
6 ~ 10 years	24.5	27.1 (2.1)	21.3 (4.7)	8.0 (0.4)
> 10 years	60.4	26.0 (3.7)	19.8 (6.7)	7.8 (0.6)
Education level				
Associate degree/teaching certificate	4.4	27.0 (3.6)	17.3 (11.0)	8.0 (0.0)
Bachelor degree	81.2	26.4 (3.2)	20.7 (6.0)	7.8 (0.6)
Master degree	14.5	25.7 (4.8)	18.5 (8.0)	7.8 (1.0)
Attended a FOYC training workshop				
Yes	86.1	25.2 (5.6)	19.6 (7.4)	7.5 (1.5)
No	13.9	27.7 (3.6)	21.5 (7.7)	8.1 (0.5)
Prior experience of teaching HIV risk reduction intervention				
Yes	12.7	25.9 (4.0)	21.4 (6.8)	7.7 (0.9)
No	87.3	26.5 (3.4)	20.1 (6.4)	7.9 (0.6)
Having other teaching priorities				
Yes	42.7	26.6 (3.6)	22.2 (5.3)*	8.0 (0.6)
No	58.2	26.3 (3.4)	18.8 (7.0)*	7.7 (0.8)
Importance of FOYC for the grade six students in your school				
Very important	90.0	26.3 (3.4)	20.1 (6.2)	7.8 (0.7)
Somewhat important	10.0	27.1 (3.4)	22.6 (8.7)	8.1 (0.7)
FOYC is a Bahamian curriculum				
Very much so	58.8	26.2 (3.0)	19.9 (6.1)	7.8 (0.6)
Somewhat	41.2	26.2 (3.8)	20.5 (6.6)	7.9 (0.8)
Performance of school coordinator				
Satisfactory	24.1	23.0 (6.5)*	16.5 (6.8)*	7.0 (1.9)*
Good/excellent	76.0	26.3 (4.8)*	20.9 (7.3)*	7.8 (1.2)*
Performance of mentor				
No mentors	51.9	24.5 (6.4)**	18.5 (7.7)**	7.3 (1.9)*
Good/satisfactory	48.1	26.7 (3.8)**	21.4 (6.8)**	7.9 (0.7)*

Note: school coordinator and mentor’s performance at school level. All other variables are at teacher level

*P < 0.05; **P < 0.10

### Bivariate Correlations Among Factors Influencing Teachers’ Implementation in Year 2

Correlations showed that teachers’ comfort level with the curriculum was significantly related to teachers’ confidence in implementing core activities (*r* = 0.59, *P* < 0.001) and attitudes toward sex education (*r* = 0.47, *P* < 0.001). Attitudes toward sex education were also significantly related to confidence (*r* = 0.34, *P* < 0.001) and perceived principal support (*r* = 0.20, *P* < 0.05). The performance of school coordinators was positively associated with the number of core activities taught (*r* = 0.26, *P* < 0.05) and the number of sessions taught (*r* = 0.25, *P* < 0.05) in the subsequent year. Number of sessions taught is highly correlated to number of core activities completed in the classroom (*r* = 0.65, *P* < 0.001; Table 2).

**Table 2:** Bivariate correlations among factors influencing teachers’ implementation in the subsequent year after the initial implementation

Variables	1	2	3	4	5	6	7	Mean	SD
1. Comfort level with the curriculum	1.00							2.36	0.55
2. Confidence in implementing core activities	0.59^c^	1.00						4.13	0.94
3. Attitudes toward sex education in schools	0.47^c^	0.34^c^	1.00					3.65	0.59
4. Perceived principal support	0.17	0.10	0.20^a^	1.00				3.70	0.60
5. Performance of school coordinators	−0.10	−0.11	−0.05	0.16	1.00			1.76	0.43
6. Performance of mentors	0.01	0.06	0.07	−0.02	0.15	1.00		0.48	0.50
7. Number of core activities taught	−0.09	−0.04	0.05	0.08	0.26^a^	0.08	1.00	25.54	5.41
8. Number of sessions taught	−0.13	0.15	−0.15	−0.05	0.25^a^	0.20	0.65^c^	7.59	1.46

Score range, 1–5 for confidence, sex education, principal support, and self-efficacy. Variables 1–4 and 7–8 are Pearson’s correlation coefficients, and 5–6 are Spearman’s correlation coefficients

*SD* standard deviation

^a^*P* < 0.05

^b^*P* < 0.01

^c^*P* < 0.001

### Mixed-Effects Models Assessing the Association Between Implementation Strategies and Teachers’ Implementation Dose in Year 2

The mixed-effects model showed that teachers’ confidence with implementing the core activities had a significant relation with the number of sessions taught (*β* = 0.17, *t* = 2.16, *P* < 0.05). The stronger predictor was “excellent/good” school coordinators on both the number of sessions taught (*β* = 0.60, *t* = 1.94, *P* < 0.05) and the number of core activities completed (*β* = 6.02, *t* = 3.21, *P* < 0.01). Number of core activities taught in the initial year was significantly associated with teacher implementation dose (core activities) in the subsequent year (*β* = 0.38, *t* = 4.42, *P* < 0.001). In addition, there was also a significant random effect of the schools on both the number of sessions taught (*β* = 0.45, *t* = 2.67, *P* < 0.01) and number of activities completed (*β* = 8.15, *t* = 2.49, *P* < 0.01), indicating that teachers at the same school, with the same school coordinators, performed similarly (Table 3).

**Table 3:** Mixed-effects model assessing the association between implementation strategies and teachers’ implementation in the subsequent year after the initial implementation

Variables	Model 1—number of sessions taught			Model 2—number of core activities completed		
	*β*	SE	*t*	*β*	SE	*t*
Fixed effect						
Education						
Associate degree	−0.181	0.316	−0.57	−0.246	1.796	−0.14
Bachelor’s degree	−0.202	0.158	−1.27	0.116	0.904	0.13
Master’s degree (ref)	0					
Comfort level with the curriculum	−0.186	0.128	−1.45	0.030	0.728	0.04
Confidence in implementing core activities	0.165	0.076	2.16*	0.701	0.435	1.61
Perceived principal support	0.041	0.089	0.46	0.309	0.509	0.61
Number of sessions or core activities completed Year 1 implementation	0.147	0.172	0.86	0.377	0.085	4.42***
Performance of school coordinators						
Excellent/good	0.599	0.309	1.94*	6.018	1.877	3.21**
Satisfactory	0			0		
Performance of site-based mentors						
Good/satisfactory	−0.020	0.294	−0.07	0.033	1.317	0.03
No mentor	0			0		
*Random effect*						
School	0.453	0.169	2.67**	8.153	3.272	2.49**

Model 1 (number of sessions taught): AIC = 285.1 for the null model and AIC = 130.8 for the final model in the table. ICC = 0.434. Model 2 (number of core activities completed). AIC = 493.6 for the null model and AIC = 341.8 for the final model. ICC = 0.325

*P ≤ 0.05, **P ≤ 0.01, ***P < 0.001

### Student Outcomes Before and After FOYC + CImPACT Curriculum in the Initial Year

Baseline Grade 6 student surveys were conducted in the classroom in the Fall of 2019. Because of the COVID-19 pandemic and school closure, administration of the classroom student follow-up survey was changed to an online format and was delayed until the beginning of Grade 7. At baseline, 2252 students completed program evaluation assessments; 850 students completed follow-up assessments. Overall, students’ HIV/AIDS knowledge, reproductive health skills, self-efficacy, and intention to use protection improved significantly from baseline to follow-up (knowledge 8.8 vs.11.2, *t* = 19.67, *P* < 0.001, Cohen’s *d* = 0.80; skills 3.8 vs. 4.6,* t* = 13.81, *P* < 0.001, Cohen’s *d* = 0.57; self-efficacy 2.5 vs. 2.8, *t* = 8.01, *P* < 0.001, Cohen’s *d* = 0.38; and intention 2.9 vs. 3.9, *t* = 14.89, *P* < 0.001, Cohen’s *d* = 0.60).

## Discussion

FOYC + CImPACT has been implemented nationwide in The Bahamas and is a global model for school-based HIV prevention programs. Program fidelity is paramount to achieve desired student outcomes, as demonstrated by previous evaluations of FOYC + CImPACT (Wang et al., 2016). With our newly developed implementation strategies, teachers in New Providence achieved high levels of initial program delivery (81% of the core activities taught) in Year 1 and continued program delivery (73% of the core activities taught) in Year 2. We observed these levels of fidelity despite a natural disaster that disrupted the education system in The Bahamas (Hurricane Dorian; Deopersad et al., 2020) and the COVID-19 pandemic, which disrupted education worldwide (Daniel, 2020). Previous research has largely focused on factors that influence the initial implementation of school-based EBI. Our study expands this literature by examining factors that affect the implementation of EBI in the subsequent year and testing two methods of enhancing implementation: use of school coordinators to provide monitoring and feedback and site-based mentorship from high-performing teachers. Although peer mentors only had a significant effect on the number of core activities taught by teachers, school coordinators’ performance with implementation monitoring and feedback was the strongest predictor of all implementation measures for initial and subsequent implementation. This support allowed for the EBI to be maintained despite the external challenges posed from 2019 to 2021.

The strategies of BMF and SAM were designed to provide opportunities for coaching, monitoring, and feedback, all of which support effective implementation (Bastable et al., 2020; Kershner et al., 2014; Wang et al., 2022). Also, there was a significant random effect of school for both the number of sessions and core activities taught, suggesting that teachers of different classes within the same school performed similarly. These teachers would have shared school coordinators, mentors, and administration personnel (e.g., principals). These combined factors supported teachers’ competency and the program fidelity. Teachers’ pre-implementation confidence in implementing the program was significantly related to the number of sessions taught. This confidence (or self-efficacy) may be bolstered by repeated trainings, BMF, and/or SAM and is consistent with previous studies of the relationship between teachers’ self-efficacy and program fidelity (Thierry et al., 2022). Teachers’ initial implementation in Year 1 was related to the number of core activities taught in Year 2. This suggests that teachers maintained their level of performance across both years of implementation. The extent to which this maintenance was due to the support provided to individual teachers (e.g., repeated trainings, school coordinators, and mentors) vs. their individual characteristics cannot be determined from these data. However, high-performing teachers were likely to remain high-performing, which is encouraging for the continuation of the curriculum for future cohorts of students.

The continued success of FOYC + CImPACT can also be attributed to several additional factors that were not quantifiable for these analyses. Contributors’ attitudes can greatly affect program fidelity. Cultural and religious considerations are vital for determining how to best approach potentially controversial topics such as safe sex practices for HIV prevention. Teachers can experience pushback when sex education programs conflict with cultural norms (Le Mat et al., 2021; Musiimenta, 2013). Webster et al. (2020) in their scoping review recommended that school administrations be more involved in collaborating with teachers and advocating for educational programs to help reduce strain from sex education opponents. FOYC + CImPACT was designed in partnership with the Bahamian MOE and Ministry of Health (MOH) to ensure that the curricula were culturally appropriate and thus would be acceptable to a majority of parents and teachers. Furthermore, Nguyen et al. (2019) reviewed the scaling-up process for reproductive health interventions that aimed to change norms surrounding sexual experiences. They summarized four factors that could serve to either inhibit or strengthen the scaling-up process and long-term implementation: financial and human resources, transferability and adaptability of intervention designs and materials, community and governmental partnerships, and the capacity to monitor or evaluate the intervention. The Bahamian MOE and MOH provided the scaffolding and support necessary to implement FOYC + CImPACT nationwide, within schools’ existing HFLE curriculum. These partnerships allow for continued access to teachers to facilitate the EBI, buy-in from school leadership, and the ability to continually monitor implementation.

Continued evaluation of FOYC + CImPACT will elucidate whether these implementation strategies will support the true sustainability of the program without external research support. There is a growing literature surrounding continued fidelity monitoring of the implementation supports that promote the sustainability of EBIs (e.g., Cook et al., 2019; Herlitz et al., 2020; Shelton et al., 2018). Improving systems that support the continued implementation of EBIs will increase the prolonged effectiveness of the programs. With FOYC + CImPACT’s inclusion in The Bahamas’ HFLE curriculum, there is a great opportunity to continue to monitor the effectiveness of the implementation supports and the program in a school setting, which will in turn provide valuable data on the sustainability of school-based programs in general.

### Limitations

This study has a few limitations. The data collected were self-reported by the teachers and thus are subject to reactivity and response bias (Smith, 2014). However, trained observers attended 10% of teachers’ sessions, and agreement between teachers and observers was high (~ 90%), providing some confidence that teachers’ self-reported data were reliable. Furthermore, this study only followed the first cohort of Grade 6 students through Grade 9 to assess long-term intervention effects; that is, we did not collect student data for the subsequent cohort. This project followed Grade 6 teachers’ implementation activities across cohorts of students to assess the program fidelity and implementation dose over time. The relation between FOYC + CImPACT program fidelity and student outcomes has been established (Wang et al., 2015, 2017) and was also observed for the first cohort of students. Thus, this analysis specifically focused on teachers’ implementation behavior in the subsequent year. In addition, the student follow-up rate is low (38%). This is because the baseline student data were collected in classrooms in September and October 2019. The follow-up student data were collected online in August 2020 after the COVID-19 pandemic caused school closures. The low follow-up rate is also due to students’ graduation from primary school and transferring into non-government middle schools (private or religious-based schools). Our student outcome evaluation is affected by the relatively low student follow-up rate.

The COVID-19 pandemic posed several challenges to FOYC + CImPACT implementation. The nature of CImPACT required parents to travel to attend the session and required the teacher to host it after typical school hours, which became difficult with the onset of the pandemic. In Year 1, only 53 of 79 teachers hosted a CImPACT session prior to school closures. For Year 2, CImPACT sessions had to be hosted virtually, which could have also posed a barrier for teachers and parents alike. Similarly, some teachers may have intended to complete the FOYC sessions in the second semester of the 2019–2020 school year but were prevented from doing so because of pandemic-related interruptions. Additionally, some FOYC core activities were not well suited for virtual instruction, and schools in The Bahamas oscillated between in-person and virtual delivery throughout Year 2. Thus, teachers were unable to teach some core activities if the lesson fell on a day with virtual school. Another impact of the COVID-19 pandemic was the difficulty in identifying and training mentors to assist teachers. Over half of the teachers (52%) did not have an assigned mentor for Year 2. Although the use of mentors has been successful in increasing teachers’ fidelity in implementing FOYC + CImPACT (Wang et al., 2022), we were unable to take advantage of those effects during this time period. However, we were able to establish and train school coordinators, who supported the teachers and increased their performances. Future qualitative data could elucidate the perceived utility of school coordinators for supporting program fidelity.

### Conclusion

Our findings contribute to a growing literature on the large-scale implementation of school-based EBI. The additional implementation strategies were robust enough to support teachers’ performances in the face of major interruptions and changes to the education system. We demonstrated that a national-scale, school-based HIV prevention EBI can be maintained with high levels of program fidelity if teachers have adequate support. These findings emphasize the importance of providing school coordinators and peer mentors who deliver ongoing support, monitoring, and feedback to increase teachers’ program fidelity. These practices, combined with rigorous training and ongoing support from the school systems, enhance the program fidelity of teachers’ implementation in the subsequent year after the initial implementation. Continued support such as BMF and SAM will allow teachers to implement EBIs with high fidelity across multiple school years, reach more students, and achieve the desired outcomes.

### Supplementary Information

Below is the link to the electronic supplementary material.

Supplementary file1 (DOCX 15 KB)

Supplementary file2 (DOCX 13 KB)

## Data Availability

Data and materials are available upon request.
