# Supplementary material for: Maintaining Program Fidelity in a Changing World: National Implementation of a School-Based HIV Prevention Program
Source: Prev Sci. 2023 Nov 18;25(3):436–47. doi: 10.1007/s11121-023-01614-1 (PMC11093787; doi:10.1007/s11121-023-01614-1)
Supplement: Supplementary file 1 — Supplementary file1 (DOCX 15 KB) [file 11121_2023_1614_MOESM1_ESM.docx]

| **Supplemental Table 1.** Implementation strategy domains | | | |
| --- | --- | --- | --- |
| **Domain** | **Strategy: Biweekly monitoring and feedback (BMF)** | **Strategy: Site-based assistance and mentorship (SAM)** | **Strategy: Annual teacher training** |
| Actor(s) | School coordinator is identified per each school as someone with a relationship and oversight over the school's HFLE teachers. Examples: lead teachers, guidance counselors, vice principals | High-performing teachers deliver>80% FOYC+CImPACT program who are trained to provide coaching and mentorship | Three Bahamian FOYC trainers and a US training specialist with extensive experience with FOYC+CImPACT for in-person training. Six high-performing Grade 6 HFLE teachers modeled the program for the virtual webinar. |
| Actions(s) | Monitors teachers’ progress regularly; provides feedback to teachers about scheduling and completing implementation checklists; notes challenges to implementation; communicates with FOYC research office; relays messages from research office to teachers | Provide coaching and mentorship to low- and moderate-performing teachers. Mentors are tained for identifying the challenges faced by teachers, assisting teachers in preparing for intervention sessions, and  providing guidance to improve curriculum delivery. | Provide a 2-day in-person training workshop and a virtual webinar for online training. Training includes curricular demonstrations of the program, didactic discussions, role play, and teach backs |
| Target(s) of the action | HFLE teachers who delivers the FOYC+CImPACT program | Low- or moderate-performing HFLE teachers | All Grade 6 HFLE teachers |
| Temporality | BMF should begin at the start of the school year | SAM should begin with the school year | Beginning of the school year during professional development time |
| Dose | Every two weeks until the entire program is completed by the teacher and all implementation checklists are submitted | As needed by target teachers | Annual 2-day in-person workshop or annual virtual webinars. |
| Implementation outcome(s) affected | Uptake of the program, delivery of the sessions, completion of implementation checklists, continued implementation of the program | Delivery of the sessions and core activities, program fidelity | Delivery of sessions and core activities, program fidelity, teacher self-efficacy and confidence in teaching the program |
| Justification | Data suggest that regular monitoring and feedback allow for timely identification and correction of implementation issues (Kershner et al., 2014) | Data support that coaching and mentorship increase program fidelity (Bastable et al., 2020) | Repeated training (Wang et al., 2015) and content-specific training (Clayton et al., 2018) are shown to increase teachers’ program fidelity of prevention programs |
