# Supplementary material for: Maintaining Program Fidelity in a Changing World: National Implementation of a School-Based HIV Prevention Program
Source: Prev Sci. 2023 Nov 18;25(3):436–47. doi: 10.1007/s11121-023-01614-1 (PMC11093787; doi:10.1007/s11121-023-01614-1)
Supplement: Supplementary file 2 — Supplementary file2 (DOCX 13 KB) [file 11121_2023_1614_MOESM2_ESM.docx]

| **Supplemental Table 2.** Missingness of the outcome and covariate variables (N=79) | | |
| --- | --- | --- |
| Outcomes | Frequency | Missing |
| Number of sessions taught | 79 | none |
| Number of core activities completed | 79 | none |
| Independent variables |  |  |
| Education |  |  |
| Comfort level with the curriculum | 71 | 8 |
| Confidence in implementing core activities | 78 | 1 |
| Perceived principal support | 77 | 2 |
| Number of sessions or core activities completed Year 1 implementation | 74 | 5 |
| Performance of school coordinators | 79 | none |
| Performance of site-based mentors | 76 | 3 |
